# Alterations in Urobiome in Patients With Bladder Cancer and Implications for Clinical Outcome: A Single-Institution Study

**DOI:** 10.3389/fcimb.2020.555508

**Published:** 2020-12-15

**Authors:** Jiarong Zeng, Guihao Zhang, Chunxiao Chen, Kun Li, Yuehui Wen, Jie Zhao, Peng Wu

**Affiliations:** ^1^ Department of Urology, Nanfang Hospital, Southern Medical University, Guangzhou, China; ^2^ Department of Urology, Meizhou People's Hospital (Huangtang Hospital), Meizhou, China; ^3^ School of Pharmaceutical Sciences, Southern Medical University, Guangzhou, China; ^4^ Clinical Microbiota Center, Nanfang Hospital, Southern Medical University, Guangzhou, China

**Keywords:** microbiome, urinary tract, recurrence, diversity, bladder cancer, outcome

## Abstract

Numerous studies indicate that resident microbiome exists in urine of healthy individuals and dysbiosis of the urobiome (urinary microbiome) may be associated with pathological conditions. This study was performed to characterize the alterations in urobiome and explore its implications of clinical outcome in male patients with bladder cancer. 62 male patients with bladder cancer and 19 non-neoplastic controls were recruited. The follow-up study cohort included 40 patients who were diagnosed with non-muscle invasive bladder cancer (NMIBC) and underwent transurethral resection of bladder tumor (TURBT). Mid-stream urine samples were collected from all the participants the day before cystoscopy. DNA was extracted from urine pellet samples and processed for high throughput 16S rRNA amplicon sequencing of the V4 region using Illumina MiSeq. Sequencing reads were filtered using QIIME and clustered using UPARSE. We found bacterial richness indices (Observed Species index, Chao1 index, Ace index; all *P* < 0.01) increased in cancer group when compared with non-neoplastic group, while there were no differences in Shannon and Simpson index between two groups. During a median follow-up time of 12 (5.25–25) months, 5/40 (12.5%)of the patients developed recurrence and no patient suffered from progression to muscle-invasive disease. Species diversity of the microbiome was significantly higher in the recurrence group compared with non-recurrence group in patients with NMIBC after TURBT. The LEfSe analysis demonstrated that 9 genera were increased (e.g., *Micrococcus* and *Brachybacterium*) in recurrence group. To our knowledge we report the relative comprehensive study to date of the male bladder cancer urinary microbiome and its relationship to pathogenesis and clinical outcomes. Given our preliminary data, additional studies evaluating the urine microbiome in relation to clinical outcomes are warranted to improve our understanding of tumor recurrence after TURBT.

## Introduction

Bladder cancer is the 7th most commonly diagnosed cancer in males (Ferlay et al., 2013). In the past decades, bladder cancer has aroused scientists’ attention for its high morbidity and recurrence rates. Unfortunately, the etiology and pathogenesis of bladder cancer is still ill-defined and clinical management remains challenging. Several important risk factors, including tobacco smoking, carcinogen exposure and genetic predisposition, may contribute to the pathogenesis of bladder cancer (Babjuk et al., 2019).

About 75% of bladder malignancies are diagnosed in superficial tumor stages (Babjuk et al., 2019). Superficial bladder cancer is characterized by high recurrence rate, with 50%–70% of patients whose bladders are not removed experiencing a relapse within 5 years of treatments (Ainsworth, 2017). The high variability in the recurrence rate indicates that the transurethral resection of bladder tumor (TURBT) was incomplete in a high percentage of patients. However, the factors that contribute to such enigmatic high recurrence rate are unknown. Because of costly lifetime surveillance with periodic cystoscopy and relevant recurrence rates, bladder cancer has the highest cost of any cancer when categorized on a per patient basis (Mossanen and Gore, 2014). This significant financial burden on the population and health care system calls for effective bladder cancer prevention and control (Fankhauser and Mostafid, 2018).

It is suggested that dysbiosis of the urobiome (urinary microbiome) may play an important role in the pathogenesis of numerous urologic diseases, such as overactive bladder and urgency urinary incontinence (Brubaker et al., 2014; Pearce et al., 2014; Pearce et al., 2015; Karstens et al., 2016; Thomas-White et al., 2016; Wu et al., 2017). Implications of urobiome in bladder cancer pathogenesis and therapeutics have also aroused abroad attention in the world (Ainsworth, 2017; Bajic et al., 2019; Markowski et al., 2019). It is reasonable to presume that dysbiosis of the urobiome may also plays a role in the pathogenesis of bladder cancer. Several lines of evidence underscored this hypothesis. Xu et al. reported some genera, such as *Streptococcus*, *Pseudomonas* and *Anaerococcus*, appearing more frequently in urothelial carcinoma patients (Xu et al., 2014). Adebayo and colleagues (2017) showed that some urinary taxa such as *Fusobacterium* and *Sphingobacterium* distinguished urogenital schistosomiasis-induced bladder pathologies from urogenital schistosomiasis infection alone and from healthy persons. It was reported that a higher abundance of *Actinomyces* was presented in bladder cancer patients, while *Bifidobacterium*, *Streptococcus*, *Lactobacillus*, and *Veillonella* were enriched in the control group (Bi et al., 2019). Liu et al. revealed higher relative abundances of the several microbial genera, such as *Acinetobacter* and *Anoxybacillus*, in the cancerous compared to the normal tissues (Liu et al., 2019). Our previous study (Wu et al., 2018) has shown increased bacterial richness, enrichment of some bacterial genera (e.g., *Acinetobacter*, *Anaerococcus*, and *Sphingobacterium*) and decrease of some bacterial genera (e.g., *Serratia*, *Proteus*, and *Roseomonas*) in bladder cancer group when compared with non-cancer group. It is worthy of note that higher abundance of the *Acinetobacter* in bladder cancer samples was also presented in several other studies (Liu et al., 2019; Mai et al., 2019). *Acinetobacter* components could play a role in carcinogenesis, by the activation of NF-κB, in animal models of bladder cancer (Roperto et al., 2012). Further studies are needed to determine the role of *Acinetobacter* as a carcinogen or co-carcinogen in humans. Hourigan et al. analyzed different urinary collection methods (voided vs. cystoscopy) in patients with bladder cancer, and revealed a difference in beta diversity in males but not in females (Hourigan et al., 2020). Pederzoli et al. define a sex-specific common bladder cancer microbiome in tissue and urine, which confirms the concordance of voided urine and tissue microbiome (Pederzoli et al., 2020). They also found *Klebsiella* was more common in the urine of female patients versus healthy controls. Of note is that *Klebsiella* have been linked with bladder cancer in another study (Mansour et al., 2020). It is postulated that the *Klebsiella* could cause direct DNA-strand damage and generate genomic instability by releasing colibactin toxin (Kaur et al., 2018). Although data linking the urinary microbiome with clinical features of bladder cancer is increasing, to date, knowledge on the relationship between them is still limited.

We hypothesized that the dysbiosis of urobiome might play a role in the pathogenesis of bladder cancer and be associated with clinical outcomes. To investigate this, we prospectively collect mid-stream urine samples from male patients with bladder cancer the day before their cystoscopy, record their clinical outcome and perform integrated analysis. Attempt was made to explore the possible association of urinary microbiome with development and clinical outcome of bladder cancer, thus provides a basis for future research.

## Materials and Methods

### Study Population and Specimen Collection

Only male participants were recruited in present study for two reasons: first, bladder cancer is male-dominated disease with almost 4.6 times as many as women (Ferlay et al., 2015); and second, there are significant gender differences of the urine microbiome (Kogan et al., 2015; Pederzoli et al., 2020). The total study cohort consisted of 62 bladder cancer, including 51 patients with non-muscle invasive bladder cancer (NMIBC) and 11 with muscle invasive bladder cancer (MIBC), and 19 non-neoplastic male patients between 2017 and 2019. All cancer cases were histologically confirmed as urothelial carcinoma and controls were cases without neoplastic conditions. All the participants were required to finish International Prostate Symptom Score (I-PSS) and those with moderate or severe symptoms (I-PSS≥8) were not eligible. To explore the role of the urinary microbiome composition in mediating clinical outcomes of patients with NMIBC after TURBT, we followed the participants after TURBT until the time to recurrence or end of follow-up. For the present study, the end of follow-up was set as September 30, 2019. Among 51 NMIBC, a total of 11 (21.6%) patients received partial cystectomy or reject surgery; they were excluded from follow-up study. Finally, 40 patients with NMIBC at initial diagnosis who underwent TURBT in a single center between 2017 and 2019 were enrolled into follow-up study cohort.

We prospectively collected Mid-stream urine samples from male patients with bladder cancer the day before their cystoscopy. Patients’ histopathological and clinical data as well as follow-up were recorded retrospectively after approval of the local ethics committee (NFEC-2020-045). All cases were re-evaluated and classified according to the recent Tumor-Node-Metastasis Classification (TNM Classification) (2019) and the WHO 2016 grading classification of genitourinary tumors by experienced uropathologists. Subjects with a recent history of sexually transmitted infection, urinary tract infections, antibiotic usage for any indication (within 1 month) were not eligible. A structured questionnaire was finished by participants to collect information on socio-demographic characteristics. Data collection followed the principles outlined in the Declaration of Helsinki. Medicine Institutional Review Board of Southern Medical University approved this study and written informed consent was obtained from all participants. About 50 ml of mid-stream urine specimens were collected by the clean catch method, and then centrifuged at 16,000 g for 10 min immediately. The pellets were stored at -80°C until further processing.

### DNA Isolation and 16S rRNA Gene Sequencing

In order to avoid contamination, DNA isolation was performed using the cultured cells protocol supplied with the DNeasy Blood & Tissue Kit (Qiagen, Germany) in a laminar flow hood. The concentration of extracted DNA was determined through a Nanodrop ND-1000 spectrophotometer (Thermo Electron Corporation, USA). The genomic DNA isolated from the clinical samples was amplified using primer sets specific for V4 regions (515F: GTGCCAGCMGCCGCGGTAA; and 806R:GGACTACHVGGGTWTCTAAT). Extraction negative controls (no urine) and PCR negative controls (no template) were included to evaluate contribution of extraneous DNA from reagents. The resultant PCR products were purified by QIAquick PCR purification kit (Qiagen, Valencia, CA). Lastly, purified samples were normalized to equal DNA concentration and sequenced using the Illumina MiSeq sequencer (Illumina, Inc., USA). The raw sequence data reported in this paper have been deposited in the Genome Sequence Archive (Genomics, Proteomics & Bioinformatics 2017) in National Genomics Data Center (Nucleic Acids Res 2020), Beijing Institute of Genomics (China National Center for Bioinformation), Chinese Academy of Sciences, under accession number CRA003441 that are publicly accessible at https://bigd.big.ac.cn/gsa.

### Bioinformatics Analysis

To obtain clean reads, raw data were filtered to eliminate reads with adapter pollution and low quality by using QIIME (Version 1.80) (Caporaso et al., 2010). Filtered sequences were clustered by 97% identity into operational taxonomic units (OTUs) using UPARSE (Edgar, 2013), and subsequently, a single representative sequence from each clustered OTU was used to align to the SILVA database (Quast et al., 2013) by Ribosomal Database Project Classifier (Wang et al., 2007).

Alpha diversity, also called within-habitat diversity, was evaluated by using QIIME, which included the Observed Species index, Chao1 index, Shannon index, Simpson index and Ace index. Among them, the Observed Species, Chao1 and Ace index represent species richness, while Shannon and Simpson index are indicators of species diversity reflecting both species richness and evenness. The difference of alpha diversity between groups was measured by Wilcoxon Rank-Sum Test (group number = 2) and Kruskal-Wallis test (n>2) using SPSS (version 22.0). Beta diversity, also called between-habitat diversity, was assessed to compare microbial composition by calculating the Bray Curtis, weighted UniFrac and unweighted UniFrac distances. Principal coordinate analysis (PCoA) was applied to generate three-dimensional plots in QIIME based on these distance matrices. The PERMANOVA was performed to test for statistical significance between groups using 999 permutations in QIIME. Differential abundance analysis between groups was performed using Metastats and *P*-values were adjusted for multiple hypotheses testing using the False Discovery Rate based on the Benjamini-Hochberg (White et al., 2009). To identify significantly different bacteria between groups, taxa summaries were reformatted and input into Linear discriminant analysis effect size (LEfSe) *via* the Huttenhower Lab Galaxy Server (Segata et al., 2011). The Kruskal-Wallis rank sum test and Wilcoxon test were used to identify biomarkers and linear discriminant analysis (LDA) was used to score them. Only taxa with logarithmic LDA score greater than 2 at a *P* < 0.05 were considered significantly enriched. ROC (receiver operating characteristic) is a graphical plot which illustrates the performance of a binary classifier system as its discrimination threshold is varied. To explore the potential ability of the microbiome to identify bladder cancer status, ROC curve analysis was constructed to analyze the clinical accuracy of using the urinary microbiome for the diagnosis and prognostication of bladder cancer.

### Statistical Analysis

Data are showed as median (first quartile to the third quartile) for continuous variables or number of cases (%) for counts data. The statistical significance of differences between groups were measured using Mann-Whitney U-test for continuous variables and Pearson’ s chi-square test or Fisher’ s Exact Test for count data through SPSS software (Version 22.0). All tests were based on two-tailed probability and *P*-value < 0.05 were considered statistically significant. PASS program (PASS 11, NCSS, Kaysville UT, USA) provided estimates of power by simulation. Estimates were obtained 2000 simulations. Kaplan-Meier survival and a univariate Cox model were was assessed using R software to investigate the correlation between microbial diversity and recurrence-free survival. We estimated that with 62 patients in cancer group and 19 subjects in control group, we would have 90% power to detect differences at the 0.05 significance level (alpha) using a two-sided Mann-Whitney U-test.

## Results

### Subjects and Samples Characteristics

A total of 99 participants were recruited, including 73 male cancer patients and 26 male non-neoplastic controls, while 11 cancer patients and seven controls were excluded for urine samples with too little sequencing reads. Therefore, the final study cohort consisted of 62 bladder cancer and 19 non-neoplastic controls (**Table 1**). Cancer group was composed of 51 patients with NMIBC and 11 patients with MIBC. No significant difference was observed in the demographic characteristics between cancer and non-cancer group, except cigarette smoking.

**Table 1:** Comparisons of demographic characteristics and parameter of alpha diversity between cancer patients and non-cancer patients.

	Cancer	Non-cancer	P-value
DEMOGRAPHIC			
Age	56.00(45.0,65.3)	46.00(35.0, 64.0)	0.07
BMI	22.85(20.76,24.50)	21.83(20.24,23.38)	0.50
Smoking habit	47(75.80)	7(36.84)	<0.01
Smoking index	550(0,800)	0(0,300)	<0.01
Drinking habit	16(25.8)	8(42.1)	0.25
Hypertension	20(32.2)	4(21.1)	0.40
Diabetes	7(11.3)	1(5.3)	0.67
CHD	3(4.8)	2(10.5)	0.33
FHC	3(4.8)	1(5.3)	0.94
ALPHA DIVERSITY			
Number of reads	115990(103454,169,274.5)	102912(61080,107378)	<0.01
Observed species	147.50(101.25,206.25)	57.0(52.00,115.00)	<0.01
Chao1	182.75(125.40,244.18)	74.50(55.33,121.00)	<0.01
Ace	191.54(132.24,248.03)	86.01(57.51,120.66)	<0.01
Shannon	2.49(1.63,3.10)	2.25(1.79,2.82)	0.42
Simpson	0.185(0.10,0.32)	0.23(0.13,0.32)	0.45

Data were presented as median (first quartile to the third quartile) for continuous variables or n (%) for counts. BMI, body mass index; CHD, coronary atherosclerotic heart disease; FHC, family history of cancer.

The follow-up study cohort consisted of 40 patients with NMIBC who underwent TURBT. The median (interquartile range [IQR]) age was 65 (52.75–69.75 years) years and median (interquartile range [IQR]) follow-up for patients was 12 (5.25–25 months) months (**Table 2**). No patient failed to follow up or fill out the surveys. After total follow-up, 5/40 (12.5%) of the patients developed a recurrence on cystoscopy, but no patient suffered from progression to muscle-invasive disease. Smoking index was higher in recurrence (RE) patients when compared with non-recurrence (NR) patients.

**Table 2:** Comparisons of clinical and histopathological parameters between Recurrence patients and non-Recurrence patients.

Characteristic	ALL	Recurrence	Non-Recurrence	P-value
Number of patients	40	5	35	–
Median age (years)	65.00(52.75,69.75)	66.0(63.50,71.00)	64.0(50.00,70.00)	0.30
Smoking habit	31(77.5%)	5(100%)	26(74.3%)	0.57
Smoking index	500.00(0.00,875.00)	800.00(800.00,2000.00)	400.00(0.00,800.00)	0.21
Grading WHO2016				
PUNLMP	10(25.0%)	0(0%)	10(28.6%)	0.30
Low grade	16(40.0%)	2(40%)	14(40.0%)	
High grade	14(35.0%)	3(60%)	11(31.4%)	
No. of tumors, n (%)				
Single	34(85.0%)	3(60%)	31(88.6%)	0.15
Multiple	6(15.0%)	2(40%)	4(11.4%)	
Stage, n (%)				
Ta	29(72.5%)	3(60%)	26(74.3%)	0.60
T1	11(27.5%)	2(40%)	9(25.7%)	
Tumor diameter				
<3 cm	31(77.5%)	2(40%)	29(82.9%)	0.07
≥3 cm	9(22.5%)	3(60%)	6(17.1%)	
Regular Epirubicin Instillation therapy				
YES	27(67.5%)	2(40%)	25(71.4%)	0.30
NO	13(32.5%)	3(60%)	10(28.6%)	

Data were presented as median (first quartile to the third quartile) for continuous variables or n (%) for counts.

### Sequencing Data, Alpha, and Beta Diversity

A total of 9,947,140 clean reads were obtained from the 81 samples. The median number of reads in cancer patients was 115,990, and in the non-cancer patients was 102,912 (**Table 1**, *P* = 0.002). The reads were classified into 3,031 OTUs used for downstream analysis. More OTUs were identified in urine from cancer patients, with an average of 165.13 OTUs per sample in cancer group and 85.74 OTUs per sample in control group (*P* = 0.0001).

**Figure 1:**
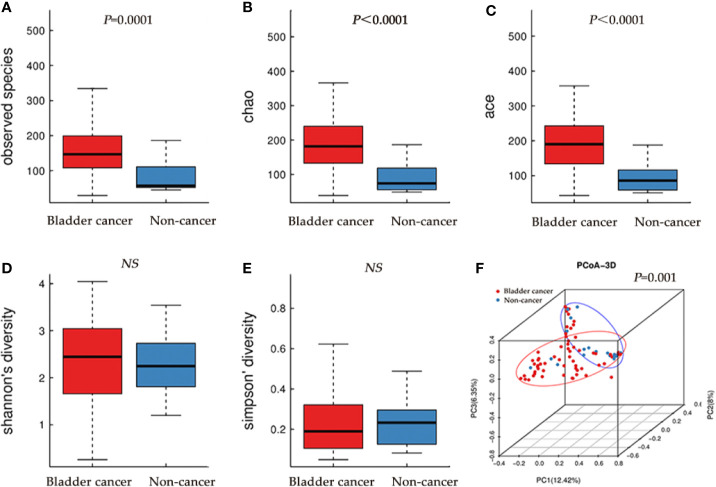
Alpha and principal coordinate analysis (PCoA) for bladder-cancer samples and non-cancer samples. **(A–E)** Box-plot showing alpha diversity in samples using different metrics (**A**, observed species index; **B**, Chao index; **C**, Ace index; **D**, Shannon index; E, Simpson index). **(F)** PCoA plots of unweighed UniFrac distances in which samples were colored by clinical outcome. The PERMANOVA performed on the Bray-Curtis distances showed that the observed differences were statistically significant (999 permutations; F = 2.71; P = 0.001).

**Figure 2:**
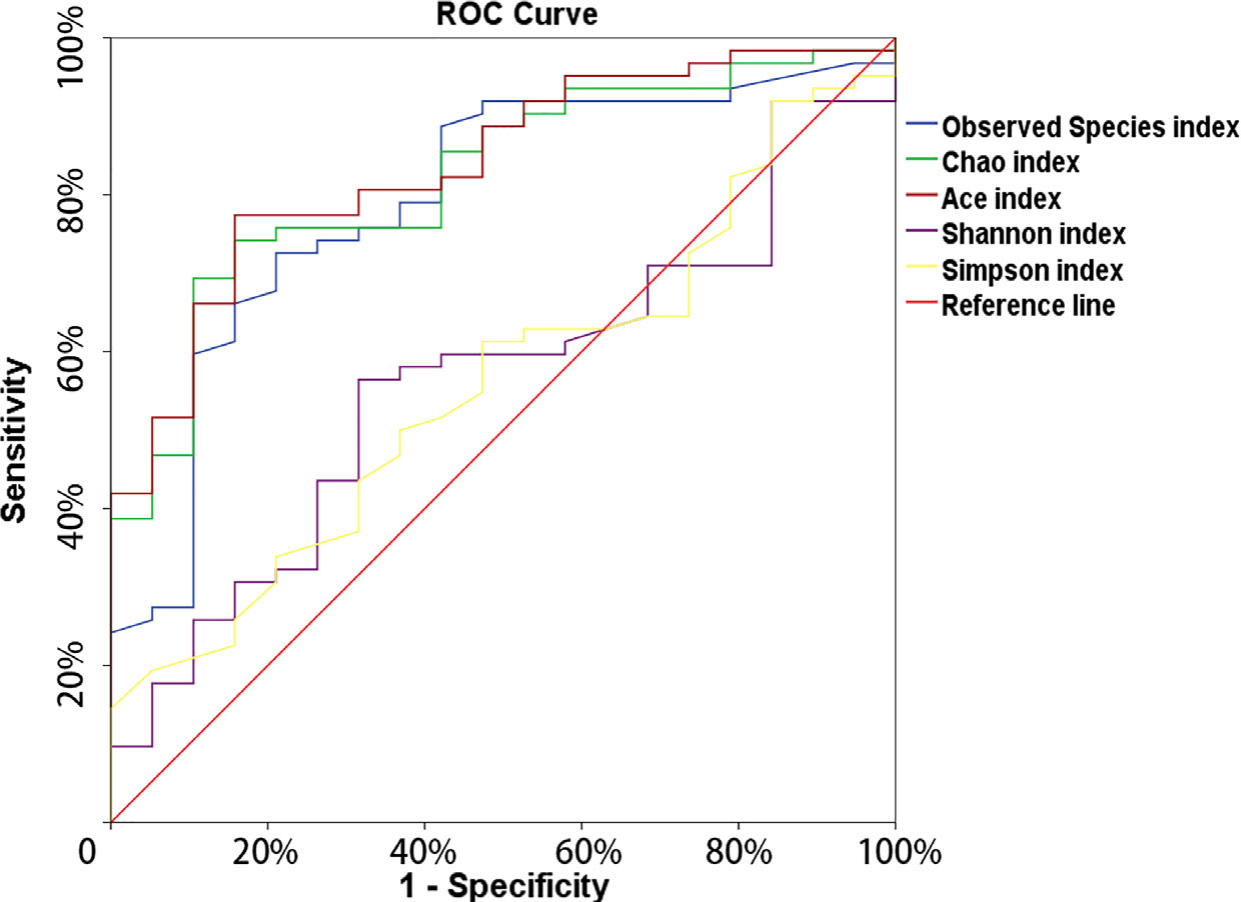
The ROC curve used to analyze the clinical accuracy of using alpha- richness obtained from the bladder-cancer group and non-cancer group for the diagnosis of bladder cancer.

The microbial richness indices (Observed Species index, Chao1 index and Ace index; all *P* < 0.01) were significantly higher in the cancer cohort than in the control cohort, while there were no differences in Shannon and Simpson index between two groups (**Figures 1A–E**, **Table 1**). PCoA revealed a clustering between OTUs from bladder cancer and control group by Bray-Curtis metric distances (**Figure 1F**), suggesting that the microbial communities exhibited phylogenetic closeness within each group (*P* = 0.001). By using ROC curve analysis, we found that AUC (area under the curve) was 0.79 (Observed Species index), 0.82 (Chao1 index) and 0.84 (Ace index) (**Figure 2**). These AUC were all above 0.70, meaning that species richness may have potential diagnostic value in patients with bladder cancer.

**Figure 3:**
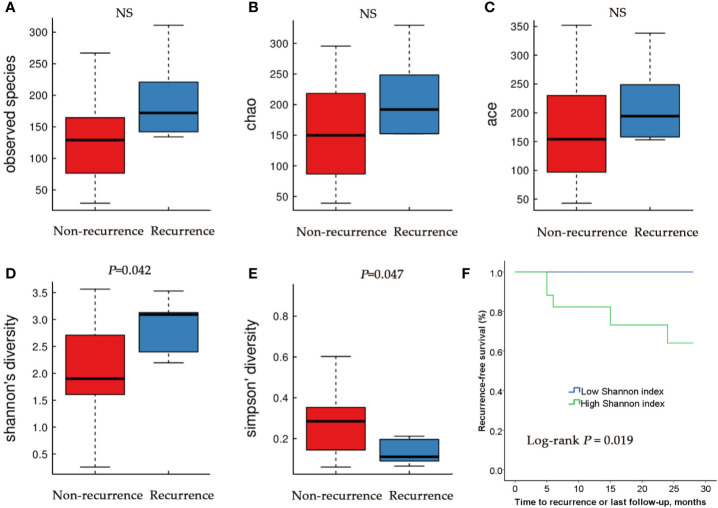
Alpha diversity and Kaplan–Meier analysis for RE patients and NR patients urinary microbiome. Observed species **(A)**; Chao index **(B)**; Ace index **(C)**; Shannon index **(D)**; Simpson index **(E)**. **(F)** Kaplan-Meier plot of patients with NMIBC defined by alpha diversity.

We then compared the microbial community of RE group and NR group. There were no differences in Observed Species index, Ace index and Chao index between RE group and NR group (**Figures 3A–C**). Higher Shannon index (diversity, *P* < 0.05) and lower Simpson index (diversity, *P* < 0.05) were represented in RE group compared with NR group (**Figures 3D, E** **)**, suggesting that RE group have higher levels of diversity.

Based on these results, we then tested the relationship between microbial diversity and clinical outcome by stratifying the patients into two groups based on median diversity (Shannon and Simpson, 2.185 and 0.21, respectively). High alpha diversity group comprised patients with above median microbial diversity, while low alpha diversity those with below median diversity. Since Shannon and Simpson index both reflect the species diversity and Kaplan-Meier test showed a similar trend of the two indexes, only one chart made by Shannon was showed (**Figure 3F**). We found that patients with low alpha diversity had significantly prolonged recurrence-free survival than those with high alpha diversity using univariate Cox proportional hazard models (*P*=0.019).

**Figure 4:**
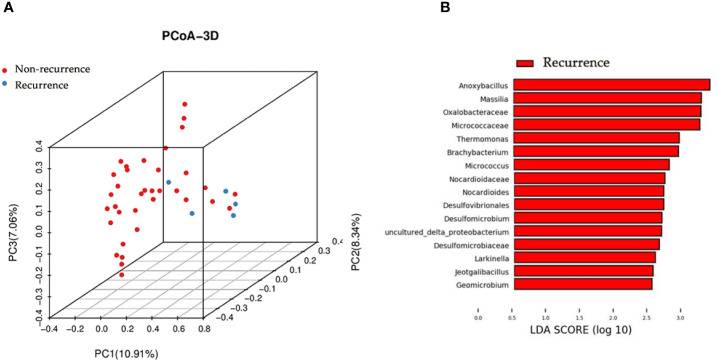
Principal coordinate analysis and LEfSe analyses for RE patients and NR patients urinary microbiome. **(A)** Principal coordinate analysis plot of the urinary microbiome based on the unweighted distance metrics. **(B)** LEfSe analyses of urinary microbiomes of RE patients compared with NR patients. Genera enriched for RE group in red. No genera enriched for NR group according to LEfSe analyses. Only genera meeting a linear discriminant analysis score threshold >2.5 are shown.

We next studied whether the microbial profile was associated with the recurrence of disease. The results showed that the microbiota composition of patients with recurrence was significantly different from that of patients without recurrence (PERMANOA, F=3.276, *P*<0.01, RE vs. NR, for unweighted UniFrac distances) (**Figure 4A**).

### LEfSe Analysis

We identified the specific taxa associated with recurrence by using LEfSe analysis. The results showed that 9 genera were overrepresented in patients with recurrence, including *Anoxybacillus*, *Massilia*, *Thermomonas*, *Brachybacterium*, *Micrococcus*, *Nocardioides*, *Larkinella*, *Jeotgalibacillus*, and *Geomicrobium* (**Figure 4B**).

### Microbial Composition Analysis

**Figure 5:**
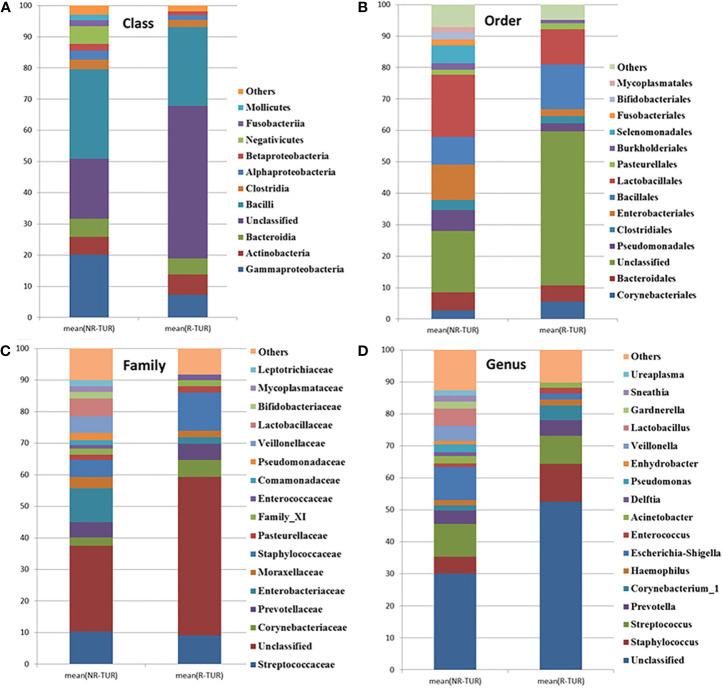
The community barplot for RE patients and NR patients urinary microbiomes. Each colored box represents a bacterial taxon and each bar, a subject. The height of a colored box represents the relative abundance of that organism within the sample. Bacterial genera with a relative abundance <1% are grouped as “Other”. Percent of community abundance on Class **(A)**, Order **(B)**, Family **(C)**, Genus **(D)** level.

The most frequently detected phylum was *Firmicutes* (28.3%RE, 37.7%NR), followed by *Proteobacteria* (10.2%RE, 25.8%NR) and *Actinobacteria*(6.7%RE, 6.7%NR) (**Table 3**). The microbial composition at class, order, family and genus level was demonstrated in **Figures 5A–D** and **Table 3**. The five most abundant class in RE group are *Bacilli*, *Gammaproteobacteria*, *Actinobacteria*, *Bacteroidia* and *Clostridia*. At the order level, *Bacillales* was the most predominant order in RE group, followed by *Lactobacillales*, *Corynebacteriales*, *Bacteroidales*, *Pseudomonadales*, and *Enterobacteriales*. The most predominant family in RE group was *Staphylococcaceae*, followed by *Streptococcaceae*, *Corynebacteriaceae*, and *Prevotellaceae*. At the genus level, *Staphylococcus* was the most predominant genus in RE group, followed by *Streptococcus*, *Prevotella*, and *Corynebacterium*_1. By using Metastats algorithm after adjusting the False Discovery Rate, no significant difference was found of relative abundance between RE and NR group at class, order, family or genus level (**Table 3**). Of note, the relative abundance of *Lactobacillus* was higher in NR group than that in RE group (5.4% vs. 0.04%), although the difference was no statistically significant.

**Table 3:** Comparison of relative abundance of urinary microbiome between NR group and RE group at all taxonomic levels.

Taxa		NR	RE	p-value	FDR
Phylum	*Firmicutes*	37.67	28.26	0.59	1.00
	*Proteobacteria*	25.75	10.23	0.02	0.53
	*Actinobacteria*	6.69	6.69	0.95	1.00
	*Bacteroidetes*	6.37	5.81	0.89	1.00
Class	*Bacilli*	28.67	25.39	0.89	1.00
	*Gammaproteobacteria*	20.13	7.34	0.01	0.53
	*Actinobacteria*	5.79	6.45	0.93	1.00
	*Bacteroidia*	5.78	5.18	0.94	1.00
	*Clostridia*	3.19	2.29	0.83	1.00
Order	*Bacillales*	8.87	14.17	0.78	1.00
	*Lactobacillales*	19.72	11.19	0.31	0.83
	*Corynebacteriales*	2.70	5.49	0.74	1.00
	*Bacteroidales*	5.77	5.18	0.94	1.00
	*Pseudomonadales*	6.70	2.64	0.09	0.63
	*Enterobacteriales*	11.24	2.21	0.03	0.63
Family	*Staphylococcaceae*	5.42	12.15	0.72	1.00
	*Streptococcaceae*	10.39	9.05	0.94	1.00
	*Corynebacteriaceae*	2.54	5.39	0.76	1.00
	*Prevotellaceae*	4.82	5.08	0.97	1.00
	*Lactobacillaceae*	5.60	0.04	0.64	0.99
Genus	*Staphylococcus*	5.17	11.89	0.76	1.00
	*Streptococcus*	10.31	8.74	0.94	1.00
	*Prevotella*	4.13	4.96	0.95	1.00
	*Corynebacterium*_1	1.64	4.54	0.80	1.00
	*Lactobacillus*	5.44	0.04	0.06	0.90

Data were reported as mean percentage; FDR, P-value after false discovery rate adjustment.

## Discussion

The present evaluation conducted urinary microbial profiling of bladder cancer *via* 16S rRNA gene sequencing. In line with previous studies (Wu et al., 2018), we found that the Observed Species index, Chao1 index and Ace index were significantly higher in cancer group when compared with non-cancer group, suggesting a higher degree of bacterial richness in cancer group. Further, we found that microbial richness might play a role in the diagnosis of bladder cancer by using ROC curve analysis.

Numerous studies have focused on the relationship between the host microbiome and cancer susceptibility or outcome in systems other than the urinary tract (Neto et al., 2015). For example, the intestinal microbiota may promote local inflammation and development of gastrointestinal cancers (Elinav et al., 2013; Garrett, 2015). Intestinal microbiome could also affect the outcomes of immunotherapeutic interventions in human cancers (Gopalakrishnan et al., 2018; Routy et al., 2018). Indirect effects of the microbiome on tumorigenesis are not well understood, and could include the modulation of antitumor immune responses (Schwabe and Jobin, 2013). Despite increasing evidence linking microbiota to cancer, the role of distal (gut) or local (urine) microbiota in urothelial carcinoma has not been fully illuminated. Urinary microbiome might create a barrier in uroepithelium, outcompete uropathogens, produce antimicrobial compounds and/or degrade harmful products (Whiteside et al., 2015).

This study makes a preliminary exploration of association of urinary microbiota and clinical feature of bladder cancer so as to promote better understanding between them. In present study, only male participants were recruited to avoid gender bias. Pederzoli et al. found distinct clustering of male versus female samples in both urine and bladder tissues, supporting the need for sex stratification in bladder cancer (Pederzoli et al., 2020). Sex related discrepancies in epidemiology, diagnosis, management, and outcomes among patients with bladder cancer have been reported (Bilski et al., 2020). It is reasonable to speculate that the difference of urinary pathogens may act an important role to gender diversities in these patients. The association of urine microbial richness with urinary health measures remains to be further explored. In the current study, we noted higher richness measures from males with bladder cancer versus those without cancer, which was in line with our previous study (Wu et al., 2018). Higher richness in the bacterial community might not be a sign of a healthy microbiome, but likely suggested the overgrowth of various harmful bacteria or archaea in patients with bladder cancer. In the Patients with non-dialysis-dependent chronic kidney disease (CKD), the diversity measures (inverse Simpson index and Chao index) were higher than those from another study that evaluated midstream voided urine samples in women without CKD (Kramer et al., 2018). Among Patients with CKD, they also noted higher diversity measures from adults with urgency urinary incontinence (UUI) versus those without UUI. In another study, more diverse urinary microbiome was reported to correlates with less robust treatment response for female UUI with an anticholinergic treatment (Thomas-White et al., 2016). Women with more diverse microbiomes either required a higher dose of the anticholinergic for the same response or did not respond to the anticholinergic at all. However, information on midstream urine microbiome diversity remain very limited.

The hypothesis of microbiome serving as a non-invasive diagnosis tool for specific diseases or cancer, including type 2 diabetes (Qin et al., 2012), hepatocellular carcinoma (Ren et al., 2019), and colorectal cancer (Yu et al., 2017), has been proposed by several studies. Our study looked at urinary microbial alterations in bladder cancer in 62 patients and 19 healthy controls, endeavoring to assess the potential diagnostic value of urinary microbial. By using ROC curve analysis, we found the AUC of microbial richness index were all approximately 80%, suggesting that microbial richness might play a potential role in the diagnosis of bladder cancer.

In the present study, we also explored the possible connection of urobiome and clinical outcomes of the NMIBC patients who underwent TURBT. Higher alpha diversity was observed in RE group when compared with NR group. Our results also demonstrated that the time to recurrence of bladder cancer differs between patients with higher alpha diversity and those with lower alpha diversity. Patients with higher alpha diversity tend to have a higher chance of recurrence compared with the men who had a lower diversity index.

Several studies have searched associations between the presence of microorganisms and clinical outcomes for specific diseases. R. Koedooder et al. (Koedooder et al., 2019) reported that vaginal microbiome profiling enables accurate prediction of both failure and success of fertility treatment. Women with an unfavorable profile had a seven times lower chance of achieving a pregnancy compared with the women who had a favorable vaginal microbiome profile. Erick Riquelme et al. (Riquelme et al., 2019) found that the tumor alpha diversity could serve as a predictor of survival outcome in resected pancreatic adenocarcinoma patients, suggesting the potential relevance of the microbiome composition in mediating pancreatic cancer progression. Urine microbial diversity may also relate to drug efficacy of patients with Urgency Urinary Incontinence (UUI) (Thomas-White et al., 2016). Compared with women with less diverse urine microbiomes, those with more diverse microbiomes either required a higher dose of the anticholinergic for the same response or did not respond to the anticholinergic at all (Thomas-White et al., 2016). These studies have preliminarily assessed the potential value of microbial composition in predicting clinical prognosis, while the specific mechanism is still unclear and it is necessary to be further verified by multicenter, prospective research.

Some bacterial taxa were richer in recurrence group by using the LEfSe analysis, such as *Anoxybacillus* and *Micrococcus.* Liu et al. also reported enrichment of *Anoxybacillus* in the cancerous tissues when compared with normal tissues (Liu et al., 2019). *Micrococcus* was recognized as an opportunistic pathogen and has been implicated in recurrent bacteremia, septic shock and endocarditis in immunosuppressed patients (Smith et al., 1999). However, their potential influence on contributing to cancer or vice versa is still unclear and more studies are necessary to determine the relationship between bladder cancer and described profiles.

Lower degree of the relative abundance of *Lactobacillus* was observed in RE group compared with NR group, although no statistically significant difference was found between two groups, which may be attributable to the low statistical power or variability within each group. The suppressor effect of *Lactobacillus* on superficial bladder cancer was an observed phenomenon but the specific mechanism is incompletely understood (Feyisetan et al., 2012). A team in Japan reported that bladder cancer patients who drank a probiotic containing *Lactobacillus casei* while also received chemotherapy treatments infused into the bladder had a 15% lower recurrence rate than those who received chemotherapy alone (Naito et al., 2008). Takahashi et al. showed that *Lactobacillus casei* intravesical therapy was superior to Bacille Calmette-Guerin (BCG) in reducing tumor growth in C3H mice bearing MBT-2 tumors (Takahashi et al., 2001). The mechanism of action by which *lactobacilli* appear to promote tumor resistance in humans has not been investigated but it has been implied that increased NK cell activity may play a role (Feyisetan et al., 2012).

To our knowledge, our study represents the first report to explore the influence of the urobiome on clinical outcomes in bladder cancer. The studies mentioned above preliminarily explored the associations between urobiome and clinical outcomes of NMIBC patients undergoing TURBT surgery, though the interaction is not clear. Our present study is also the relative comprehensive study in which profiling the urinary microbiome associated with bladder cancer to date. Nevertheless, there are several limitations as follows.

Firstly, the precision of our estimates is still limited by a moderate sample sizes and follow-up time. As such, we have now determined the sample sizes and follow-up time needed to further evaluate the relationships between microbiome and bladder cancer in our future larger studies. Secondly, we collect the samples only once and prior to the surgery in our study, so we cannot comment on microbial stability over time. Meanwhile, account must be taken for the outcomes of patients undergoing surgery depends on multiple clinical parameters (e.g. tumor factors, operation factors, bladder instillation regimen). Prospective studies are needed to disentangle the association between cancer development and microbial dysbiosis, as well as the possible role of these bacterial communities in the metabolism of carcinogenic compounds present in the urinary tract (Alfano et al., 2016). Moreover, the generalizability of predictive model is also needed to be proved in the external validation cohort. Last but not least, despite the use of voided samples avoids the participant burden of urinary catheterization, microbial assessments obtained using voided samples may include microbial contributions from adjacent pelvic niches (Kramer et al., 2018).

## Conclusion

In conclusion, our study suggests that urobiome may be associated with bladder cancer and clinical outcomes of patients with NMIBC after TURBT. Given our preliminary data, we think that additional studies evaluating the urine microbiome in relation to clinical outcomes are warranted and hold promise to improve our understanding of disease recurrence after TURBT. The clinical applicability of these findings has not yet been integrated into daily practice. Developing a predictive test based on the urobiome composition will contribute to a personalized medicine approach in the future.

## Data Availability Statement

The datasets presented in this study can be found in online repositories. The names of the repository/repositories and accession number(s) can be found in the article/supplementary material.

## Ethics Statement

The studies involving human participants were reviewed and approved by Medicine Institutional Review Board of Southern Medical University. The patients/participants provided their written informed consent to participate in this study.

## Author Contributions

Conception and design: JZe, GZ, PW. Acquisition of data: JZe, GZ, CC, KL. Analysis and interpretation of data: JZe, JZh. Drafting of the manuscript: JZe, GZ. Critical revision of the manuscript for important intellectual content: PW, JZh, YW. Statistical analysis: PW, JZh. Obtaining funding: PW. Supervision: PW. All authors contributed to the article and approved the submitted version.

## Funding

This study is supported by the Natural Science Foundation of Guangdong Province (grant no. 2018A030313148), the Natural Science Foundation of Guangdong Province (grant no. 2020A1515011339) and the National Natural Science Foundation of China (grant no. 81870522).

## Conflict of Interest

The authors declare that the research was conducted in the absence of any commercial or financial relationships that could be construed as a potential conflict of interest.
